# Discovery of lignin-transforming bacteria and enzymes in thermophilic environments using stable isotope probing

**DOI:** 10.1038/s41396-022-01241-8

**Published:** 2022-05-02

**Authors:** David J. Levy-Booth, Laura E. Navas, Morgan M. Fetherolf, Li-Yang Liu, Thomas Dalhuisen, Scott Renneckar, Lindsay D. Eltis, William W. Mohn

**Affiliations:** 1 Department of Microbiology and Immunology, Life Sciences Institute, BioProducts Institute, The University of British Columbia, Vancouver, BC Canada; 2 Advanced Renewable Materials Lab, Department of Wood Science, BioProducts Institute, The University of British Columbia, Vancouver, BC Canada

**Keywords:** Water microbiology, Stable isotope analysis

## Abstract

Characterizing microorganisms and enzymes involved in lignin biodegradation in thermal ecosystems can identify thermostable biocatalysts. We integrated stable isotope probing (SIP), genome-resolved metagenomics, and enzyme characterization to investigate the degradation of high-molecular weight, ^13^C-ring-labeled synthetic lignin by microbial communities from moderately thermophilic hot spring sediment (52 °C) and a woody “hog fuel” pile (53 and 62 °C zones). ^13^C-Lignin degradation was monitored using IR-GCMS of ^13^CO_2_, and isotopic enrichment of DNA was measured with UHLPC-MS/MS. Assembly of 42 metagenomic libraries (72 Gb) yielded 344 contig bins, from which 125 draft genomes were produced. Fourteen genomes were significantly enriched with ^13^C from lignin, including genomes of *Actinomycetes* (*Thermoleophilaceae*, *Solirubrobacteraceae*, *Rubrobacter* sp.), *Firmicutes* (*Kyrpidia* sp., *Alicyclobacillus* sp.) and *Gammaproteobacteria* (*Steroidobacteraceae*). We employed multiple approaches to screen genomes for genes encoding putative ligninases and pathways for aromatic compound degradation. Our analysis identified several novel laccase-like multi-copper oxidase (LMCO) genes in ^13^C-enriched genomes. One of these LMCOs was heterologously expressed and shown to oxidize lignin model compounds and minimally transformed lignin. This study elucidated bacterial lignin depolymerization and mineralization in thermal ecosystems, establishing new possibilities for the efficient valorization of lignin at elevated temperature.

## Introduction

Lignin is the second most abundant terrestrial biopolymer after cellulose, and can comprise 10–30% of a plant’s dry weight [1]. While biological conversion of plant biomass to fuels and chemicals can reduce fossil fuel consumption, few processes exist to valorize lignin to value-added chemicals [2]. This is due in part to its recalcitrance and heterogeneity: lignin is a complex heteropolymer containing diverse ether and carbon-carbon bonds linking phenylpropanoid aromatic subunits. A promising approach to divert lignin from waste streams for production of valuable bio-products is biological lignin valorization, involving lignin depolymerization and biocatalysts that funnel lignin-derived aromatic compounds (LDACs) into commercial chemicals [2]. There is a need to develop thermotolerant biocatalysts for efficient conversion of lignin derivatives produced by industrial processes [3].

In nature, fungi are thought to be mainly responsible for lignin depolymerization, with white rot fungi utilizing lignin peroxidases (EC 1.11. 1.14) and laccases (benzenediol oxygen oxidoreductases, EC 1.10.3.2) to do so. However, bacteria are increasingly recognized for their contributions to this process [4–6]. Investigation of bacterial lignin depolymerization has focused on two enzyme classes: dye-depolymerizing peroxidases (DyPs) and laccase-like multi-copper oxidases (LCMOs). Bacterial LCMOs carry out myriad reactions. Importantly, LCMOs in two-domain super-families, including K-type small laccases (SLACs), are capable of efficient lignin depolymerization [7, 8]. Further, bacteria have catabolic pathways that funnel diverse LDACs into catabolic intermediates (e.g., protocatechuate and catechol). These diols are typically degraded via either *meta* or *ortho* ring-cleavage pathways [6, 9]. Characterization of novel thermotolerant lignin-degrading bacteria therefore requires (1) evidence for their involvement in lignin degradation, (2) identification of enzymes that depolymerize lignin, and (3) identification of pathways for catabolic funneling of LDACs.

To facilitate identification of novel lignolytic organisms and biocatalysts, we undertook genomic bioprospecting in thermal environments. Massive piles of wood residue, known as “hog fuel,” can reach temperatures sufficient for spontaneous combustion due to biological activity. We hypothesized that thermophilic microbes within hog fuel are adapted to use lignin and LDACs as carbon sources. Likewise, we hypothesized that geothermal hot springs with regular inputs of woody biomass harbor thermophilies capable of catabolizing lignin. To test these hypotheses, samples of hog fuel and hot spring microbial communities were incubated with a synthetic ^13^C-ring-labeled lignin dehydrogenation polymer (DHP) to facilitate stable-isotope probing (SIP). ^13^C-Enriched genomes resolved from metagenomic libraries encoded a variety of enzymes with the potential for depolymerization of lignin and catabolism of LDACs. Subsequently, we heterologously expressed a two-domain LMCO and characterized its ability to transform β-aryl ether lignin model compounds and Eucalyptus milled wood lignin.

## Materials and methods

### Sampling thermal environments

Lakelse hot spring (54°21′30.7″N, 128°32′28.0″W), near Terrace, Canada, is a concrete-enclosed pool 1–5 m deep, fed by geothermally warmed spring water (53.2 °C, pH 7.5) [10, 11]. About 500 ml of the top ~5 cm layer of organic-rich sediment was sampled at four equidistant locations on August 24th, 2017 using a manual pump. Sediment and spring water were placed in autoclaved 1 L Nalgene bottles and placed on ice. Two additional sediment samples per location for DNA extraction were placed in 5 ml screw-top vials and placed immediately on dry-ice. A 1.93 ha hog fuel pile in Crofton, Canada (48°52′31.9″N, 123°39′08.1″W) containing sub-boreal spruce, western redcedar and Douglas-fir reside was sampled on September 27, 2017. Three 1 m pits were dug at 40 m intervals along the perimeter of the pile and ~500 ml samples were removed from 20 and 80 cm depths (52.9 °C and 58.7 °C, respectively) with an ethanol- and distilled H_2_O-washed trowel. Bulk samples were stored on ice and 5 ml aliquots were stored on dry ice.

### ^13^C-DHP lignin microcosms

Sediment was separated from spring water using Steritop Filter bottles (Sigma-Aldrich, St. Louis, U.S.A.). Two sets of three replicate sediments from Lakelse, and hog fuel samples from 20 and 80 cm equivalent to 1 g dry weight were added to autoclaved 50 ml serum bottles with 0.1 g of 10% ^13^C-DHP lignin or ^12^C-DHP lignin plus 5 ml M9 buffer [12], and the bottles were crimp-sealed. ^13^C-DHP lignin was synthesized as in [4] and in the Supplementary Methods. Lakelse sediment and 20 cm hog fuel were incubated at 53 °C, while 80 cm hog fuel was incubated at 62 °C. Incubations were in rotary shakers at 150 rpm.

### ^13^C-CO_2_ respiration analysis

We monitored ^13^C-CO_2_ production as an indicator of ^13^C-DHP lignin mineralization. In total, 0.5 ml of serum bottle headspace air was manually-injected into an Isoprime gas chromatograph isotope ratio mass spectrometer (GV Instruments, Wythenshave, U.K.) using a 1.0-ml glass syringe. Headspace CO_2_ concentrations were calculated using a standard curve of 0.05, 0.5, 5, and 10% of ^12^C-CO_2_ (~1.2% atom ^13^C Praxair Inc., Danbury, U.S.A.) in N_2_, and 99.0 atom % ^13^C CO_2_ (Sigma-Aldrich) in N_2_ to 0.01, 0.05, 0.1 and 0.5% ^13^C-CO_2_. Control microcosms without sediment were monitored to test stability of ^13^C-DHP lignin at elevated temperature.

### DNA extractions and fractionation

DNA was extracted from 0.5 g of three replicate in situ thermal hot spring sediment and hog fuel samples using NucleoSpin Soil kits (Macherey-Nagel, Düren, Germany). After 24 days incubation, three replicate microcosms with each of the three inocula were emptied into sterile 15-ml Falcon tubes and centrifuged for 10 min at 4000 rpm at 4 °C. DNA was extracted 4 times from 0.5 g sediment or hog fuel using the above kit to achieve ≥5.0 µg recovered DNA. Cesium chloride density gradient centrifugation and fractionation was conducted according to published protocols [13, 14]. The level of ^13^C enrichment in each purified DNA fraction was quantified using ultrahigh-performance liquid chromatography-tandem mass spectrometry (UHPLC-MS/MS). Details are provided in [15] and in the Supplementary Methods.

### Fraction selection and shotgun sequencing

Fraction four (F4, ~1.737 g ml^−1^) was selected as the “heavy” fraction, based on density measurement, % atom ^13^C-DNA, and absence of DNA recovered in this fraction from ^12^C-DHP lignin microcosms. Fraction six (F6, ~1.727 g ml^−1^) was used as the heavy fraction for ^12^C-DHP lignin samples. Fraction ten (F10, ~1.717 g ml^−1^) was used as the “light” fraction for both. One ng DNA from light and heavy fractions were used to generate metagenomic sequencing libraries using the Nextera XT DNA Library Prep Kit (Illumina, San Diego, U.S.A.). In total, 12 libraries from hog fuel microcosms and 12 libraries from hot spring microcosms (Supplementary Table 1) were multiplexed separately on two runs of NextSeq (Illumina) using 150-bp paired-end sequencing in High Output mode at the UBC Sequencing and Bioinformatics Consortium (Vancouver, CAN).

### Metagenome assembly, binning and annotation

Trimmomatic 0.36 [16] was used to quality filter reads and trim Illumina adapters using default parameters. Reads from hot spring and hog fuel libraries were assembled separately with metaSPAdes v3.11.1 [17] using kmers = [21,33,55,77,99,127]. Contigs were binned with MyCC [18], MetaBAT2 2.12.1 [19], CONCOCT 1.0 [20] and MaxBin2.2.7 [21], and a dereplicated set of metagenome-assembled-genomes (MAGs) was generated with DASTool 1.1.2 [22]. MAGs were assessed for completeness and redundancy using CheckM 1.0.1 [23]. Taxonomic classification used GTDB-Tk [23] (github.com/Ecogenomics/GTDBTk). Coding sequences in binned and unbinned contigs were predicted using Prodigal 2.6.3 [24], and annotated with (1) DIAMOND 0.9.22.123 [25] blastp against the RefSeq 94 non-redundant (nr) database with a cut-off of *e* ≤ 1E−50, (2) hidden Markov models (HMMs) against the carbohydrate-active enzymes (CAZy) [26] database with dbCAN2 [27], Pfam/TIGRfam [28, 29], and (3) the KEGG database using the HMM-based KOFAMSCAN [30].

### Statistical analysis

Base-2 logarithmic fold change (L_2_FC) of MAG abundance (sequencing depth) between ^13^C heavy (“enriched”) and light fractions, as well as between ^13^C heavy and ^12^C heavy fractions, was determined with *DeSeq2* [31] in R 3.5.1 [32]. Phylogenetic trees were visualized using iTol [33] and *ggtree* [34].

### Laccase preparation and characterization

LacO_ST51_, identified in an enriched MAG from hog fuel, was produced heterologously as an N-terminal polyHis-tagged (Ht-) protein using *E. coli* BL-21 λ (DE3) containing pET_LacO_ST51_ (details in Supplementary Methods). The molecular weight and purity of the protein were analyzed using SDS-PAGE. The copper content of LacO_ST51_ was quantified using 2,2′-bicinchoninic acid after reduction of copper ions released from the holoenzyme [35]. Laccase activity was measured spectrophotometrically at 436 nm (*ε*  =  36,000 M^−1^ cm^−1^) and 468 nm (*ε*  =  49,600 M^−1^ cm^−1^) for assays performed using 3 mM 2,2’-azino-bis(3-ethylbenzothiazoline-6-sulfonic acid) (ABTS) (20 mM sodium acetate, pH 5) or 1 mM DMP (20 mM sodium phosphate, pH 8), respectively. One unit of activity (U) is defined as the amount of enzyme required to transform 1 μmol of substrate to product per minute at 25 °C. The specific activity of sLac from *Amycolatopsis* sp. 75iv3, a SLAC (Singh et al. [7]), was determined in parallel under the same conditions as a positive control. The optimal pH of LacO_ST51_ for DMP was evaluated over a range between pH 6 and 9 using 20 mM sodium phosphate (*I* = 0.1 M, pH 6–8) and 20 mM Tris-HCl (*I* = 0.1 M, pH 9) buffers. The thermostability of the enzyme was analyzed by measuring the residual activity on DMP at pH 8, after incubating the enzyme at 45, 55, 65 and 75 °C for up to 24 h.

The ability of LacO_ST51_ to transform guaiacylglycerol-β-guaiacyl ether and veratrylglycerol-β-guaiacyl ether was performed as described elsewhere [36]. Briefly, 1 mM of β-*O*-4 biaryl ether was incubated with 1 μM LacO_ST51_ in 20 mM sodium phosphate, pH 8. The reactions were incubated at 55 °C with stirring, and quenched after 6 h by adding acetic acid to 10% final concentration. The quenched reaction was centrifuged at 16,000 × *g* for 5 min, and the cleared solution was analyzed by reverse-phase HPLC.

To characterize activity with a minimally transformed lignin, enzymatic mild acidolysis lignin (EMAL) from Eucalyptus wood [37] was dissolved in DMSO (100 mg ml^−1^) and used at 0.5% (w/v) for assays. Reactions were in performed in 10 ml 12.5 mM potassium phosphate, pH 8, containing 10% DMSO, incubated with or without 6 μM laccase at 30 °C and 200 rpm for 6 days. Reactions were performed using either LacO_ST51_ or sLac from *Amycolatopsis* sp. 75iv3. To analyze the release of monomers after incubation, 100 μl of each reaction was quenched by adding acetic acid to 10% final concentration and analyzed by reverse-phase HPLC. The dried lignin was further analyzed by HSQC NMR and gel permeation chromatography (GPC). Full assay details are provided in Supplementary Methods.

## Results

### ^13^C-lignin catabolism

**Fig. 1:**
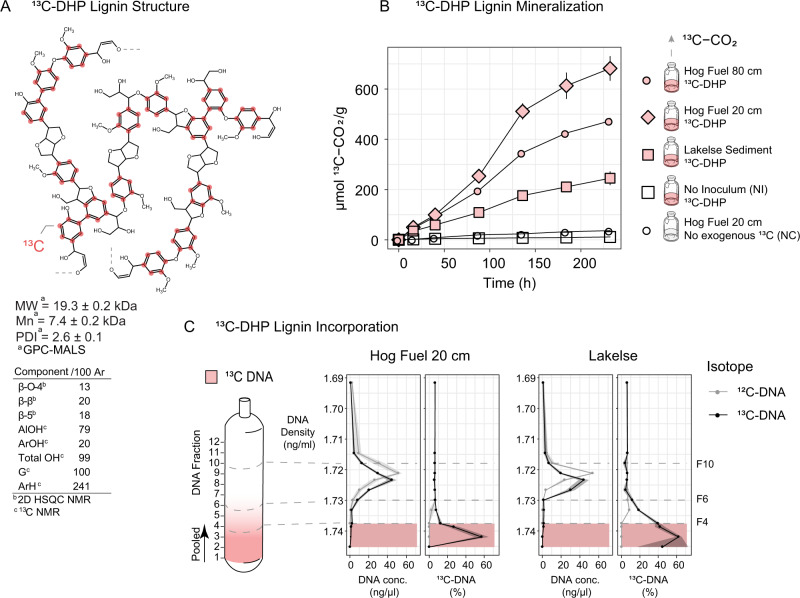
Stable isotope probing (SIP) using ^13^C-DHP lignin polymer. **A** Detail of DHP lignin structure supported by GPC-MALS, 2D HSQC NMR and ^13^C NMR analysis. Mn number average molecular weight (average molecular weight of all the polymer chains), PDI polydispersity index or Mw/Mn ratio. Mw, Mn and PDI determined by GPC-MALS. Red dots show possible positions of ^13^C-isotopes in coniferyl subunit rings. **B** ^13^C-CO_2_ measured in the headspace of 50-ml sealed serum bottles containing 1 g (dw) of each sample incubated with 100 mg (10%) ^13^C-DHP lignin, determined by IR-GCMS. ^13^C-CO_2_ evolution for control bottles with no inoculum (NI) or 100 mg unlabeled ^12^C-DHP lignin (NC) are provided. Each point is the mean of *n* = 3, and error bars represent standard error. **C** Post-ultra-centrifugation DNA gradient in a 5 ml centrifuge tube with ^13^C-Low (F10), ^12^C-Low (F10), ^13^C-High (F4) and ^12^C-High (F6) demarcated with dashed gray lines. Graphs show DNA concentration vs. fractional density and %^13^C-DNA for Hog Fuel 20 cm and Lakelse samples. %^13^C-DNA measured using UPLC-MS/MS.

In this study, we investigated the ability of thermophilic bacteria to mineralize synthetic lignin and assimilate lignin derivatives via stable isotope probing. We first synthesized ~3.5 g ^13^C-DHP with a mean molecular weight of 19.3 ± 0.2 kDa, approximately equivalent to 100 aromatic nuclei per DHP polymer. This synthetic lignin contained 13 β-O-4, 20 β-β, and 18 β-5 bonds per 100 guaiacyl subunits (Fig. 1A).

Approximately 0.75% (w/w) ^13^C-DHP or ^12^C-DHP (control) was incubated with 2 g hot spring sediment or ground hog fuel for up to 24 days. We monitored the mineralization of ^13^C-DHP to ^13^C-CO_2_ to determine if the lignin was mineralized (Fig. 1B). Measuring the incubation headspace using IR-GC-MS showed that, after 24 days, hog fuel from 20 and 80 cm depths evolved about 473 ± 18 and 681 ± 82 µmol ^13^C-CO_2_ per gram of hot spring sediment or ground hog fuel, respectively, while Lakelse sediment evolved about 245 ± 45 µmol ^13^C-CO_2_ g^−1^. For comparison, ^13^C-DHP incubated without inoculum evolved 12 µmol ^13^C-CO_2_ g^−1^, and sediment incubated without ^13^C-DHP evolved 36 µmol ^13^C-CO_2_ g^−1^.Thus, microbial communities from thermal environments mineralized lignin at in situ temperatures.

Density fractionation was used to isolate DNA from microbes that incorporated ^13^C from the labeled synthetic lignin during incubation. To verify isotopic-labeling of DNA, UPLC-MS/MS was used to calculate atom% ^13^C in each of the 12 recovered fractions (Fig. 1C). Fractions 1–4 (F1–4) from ^13^C-DHP microcosms had a mean density of 1.738–1.745 g ml^−1^ and contained about 20–60 atom% ^13^C. All fractions from ^12^C-DHP microcosms had a baseline of about 6 atom% ^13^C DNA. Thus, fractions 1–4 were pooled to recover sufficient ^13^C-DNA for shotgun metagenome sequencing. Detectable DNA was not recovered from fractions 1–4 of the ^12^C-DHP microcosms. Therefore, fraction 6 (F6; 1.72 g ml^−1^) was used for the “high-density” fraction for these control microcosms.

### Resolution of genomes from ^13^C-enriched metagenomes

**Fig. 2:**
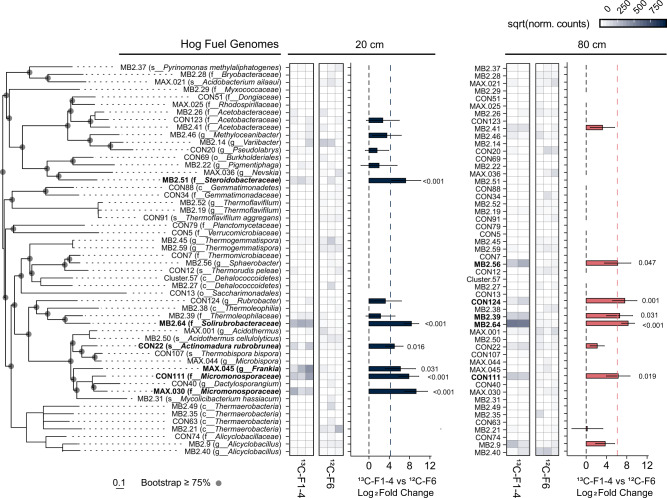
Hog fuel MAG abundance in ^13^C-High (F1–4) and ^12^C-High (F6) SIP libraries. Phylogenetic tree MAG placement using GTDB-TK v1.0.2 based on 120 bacterial single-copy genes. ^13^C-enriched genomes shown in bold. Scale shows length equivalent to 0.1 substitutions. Heatmap shows square-root transformed mean MAG abundance following DeSeq2 normalization in triplicate libraries. Abundance calculated by mapping quality-filtered reads to MAG nucleotide sequences with bbmap 38.22. Bar plot shows log_2_ fold change (L_2_FC) between ^13^C-High and ^12^C-High for each genome with >L_2_FC 0 indicating enrichment in ^13^C-High libraries. Error bars represent standard error of L_2_FC. Bar plot provides cut-off estimates for significance at *α*_adj_ = 0.05 (individual *p*_adj_ values <0_._05 provided).

**Fig. 3:**
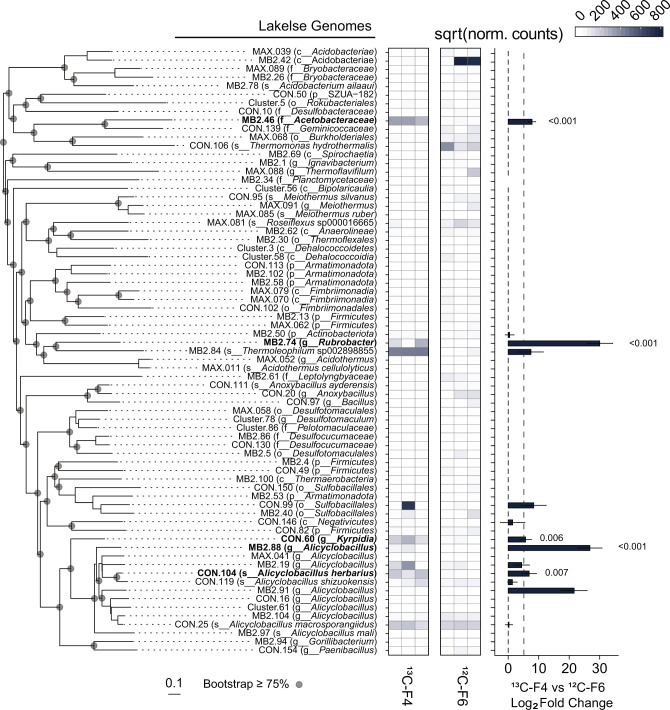
Lakelse MAG abundance in ^13^C-High (F1–4) and and ^12^C-High (F6) SIP libraries. Phylogenetic tree, heatmaps, and bar plot as in Fig. 2.

To facilitate the identification and characterization of putatively lignolytic bacteria in thermal environments, we focused our investigation on genome assemblies resolved from shotgun sequencing of fractionated DNA. Fifty-two of these MAGs passing quality thresholds (>80% completion, <5% contamination) were assembled from hog fuel and 72 from hot spring sediment. DESeq2 was used to statistically compare MAG abundance between high-density fractions from ^13^C-DHP (F1–4) and ^12^C-DHP (F6) microcosms (Figs. 2 and 3), as well as between high-density and low-density (F10) fractions from ^13^C-DHP microcosms (Figs. S2 and S3). MAGs with significantly higher (*p*_HDR_ < 0.05) abundance in ^13^C F1–4 relative to ^12^C F6 were considered ^13^C-enriched. There were four ^13^C-enriched MAGs in hog fuel 20 cm libraries, another three in the 80 cm libraries, and two in libraries from both depths. There were five ^13^C-enriched MAGs in hot spring sediment libraries. One gammaproteobacterial MAG enriched in hog fuel (MB2.51) was placed in family *Steroidobacteraceae* (Fig. 2). The remainder of enriched MAGs were Gram-positive bacteria, including the phyla *Chloroflexi*, *Actinobacteria* and *Firmicutes*. MB2.64 from hog fuel was placed in the thermophilic actinobacterial family *Solirubrobacteraceae*, and was enriched over 300-fold in both 20 and 80 cm libraries.

**Fig. 4:**
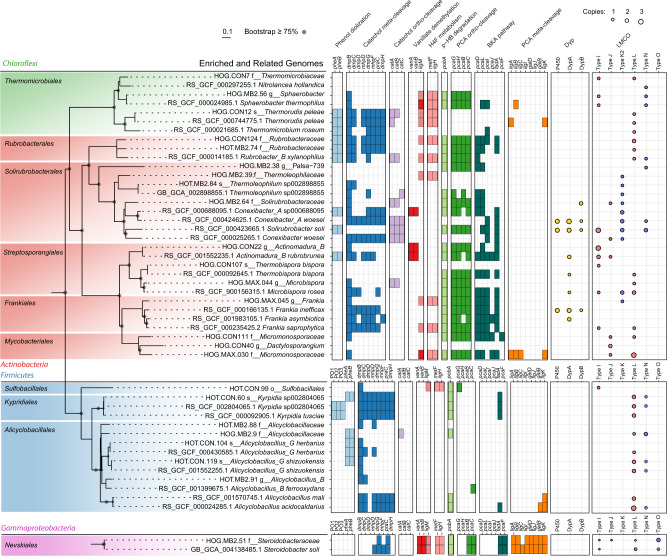
Predicted aerobic aromatic degradation pathways, dye-depolymerizing peroxidases (Dyps) and laccase-like multi-copper oxidases (LMCOs) in ^13^C-DHP lignin enriched MAGs and select reference genomes. Phylogeny of MAGs as in Figs. 2 and 3. Aromatic degradation pathway genes were annotated using profile HMMs for KEGG orthologs (KO) with *e* < 0.01 and HMM scores above KO-specific thresholds. Syringate O-demethylase (LigM) orthologs annotated using the TreeSAPP reference package are shown with 50% opacity (i.e., light pink). Individual orders containing enriched MAGs are highlighted. H4F Tetrahydrofolate, PCA Protocatechuic acid, HB Hydroxybenzoate, BKA beta-ketoadipate.

While we were able to recover 125 MAGs with an average single-copy gene completeness of 87% (Supplementary Data 2), the full suite of metabolism-encoding genes was likely not recovered for all, potentially resulting in incomplete annotation of aromatic degradation pathways. Of the hog fuel MAGs, MB2.64 (99% completeness), encoded catechol and protocatechuate *ortho*-cleavage pathways, and 4-hydroxybenzoate monooxygenase (Fig. 4). A *Thermoleophilaceae* MAG, MB2.39 was also highly enriched with ^13^C in the 80 cm hog fuel microcosms, and like MB2.64, is a member of the thermophilic order *Solirubrobacterales*. Other ^13^C-enriched *Actinobacteria* include CON22 (*Actinomadura rubrobrunea*), MAX.045 (*Frankia* sp.), MAX.030 (*Micromonosporaceae*), CON124 (*Rubrobacter* sp.) and MB2.74 (*Rubrobacter* sp.) (Fig. 3). These MAGs all encoded protocatechuate degradation. In addition, *A. rubrobrunea* encoded a two-component vanillate *O*-demethylase, while the *Micromonosporaceae* MAG appeared to encode a LigM-type (aminomethyltransferase) vanillate *O*-demethylase based on pHMM results (Fig. 4). To account for incomplete annotation of our MAGs, their aromatic degradation pathways were compared with those annotated in closely related strains (Fig. 4), revealing that vanillate *O*-demethylation and protocatechuate *ortho*-cleavage are encoded widely in thermophilic *Actinobacteria*.

The ^13^C-enriched Lakelse hot spring MAGs represented a higher proportion of *Firmicutes* than the hog fuel MAGs (Figs. 2 and 3). These included *Kyrpidia* sp. (CON.60) and *Alicyclobacillus* sp. (MB2.88, CON.104). While some *Kyrpidia* and *Alicyclobacillus* reference genomes encode catechol *meta*-cleavage (Fig. 4), only CON.60 was found to encode this pathway in our MAG dataset. Three *Alicyclobacillus* genomes (MB2.88, CON25, and MB2.97) encoded LMCOs with high amino acid identity (>85%) to a homolog in phenolic- and polyphenolic-oxidizing *Alicyclobacillus acidocaldarius* DSM 446 [38]. Of these, MB2.88 contained two L-type 3-domain LCMOs with 100% amino acid identity to those encoded by DSM 446.

### Identification of putative ligninases

**Fig. 5:**
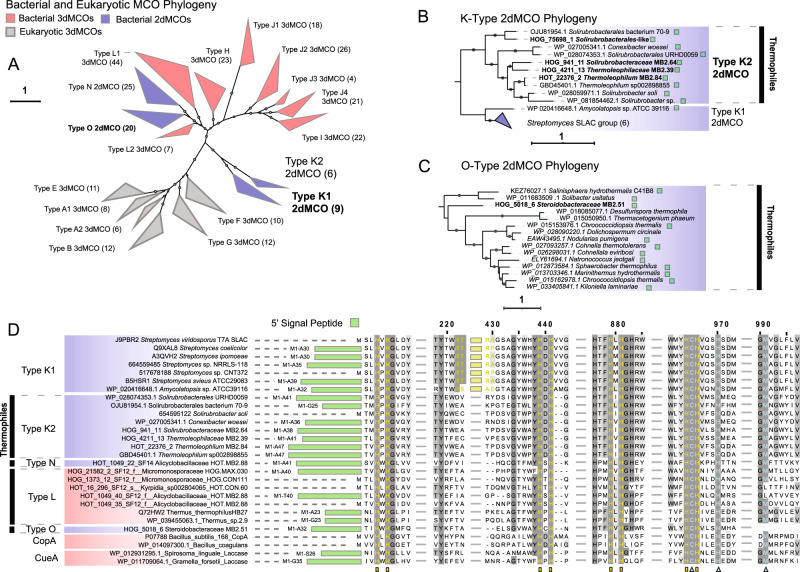
Laccase-like multi-copper oxidase (LCMO) phylogeny and classification using TreeSAPP LCMO reference package. **A** Phylogeny of reference LCMOs. Protein sequences were aligned using MAFFT using the ginsi setting under 1000 iterations. Phylogeny was reconstructed using RAxML under the PROTGAMMAPMB model with 1000 iterations. Values beside labels show the number of reference sequences for each clade. **B** K-type (2dMCO SLAC) sequences from MAGs placed into reference tree. Presence of 5’ signal peptides shown using a green square. **C** Phylogenetic placement of LCMO sequences from MAGs in the O-type (2dMCO) clade. **D** Multiple alignment of reference and MAG LMCO sequences. Blue denotes 2dMCOs and red denotes 3dMCOs. Length of 5’ signal peptides shown in green. Yellow markings denote conserved copper-binding residues. Blue markings denote substrate-binding residues. Gray denotes strength of conservation. Yellow region shows possible active-site protecting fold in Type-K1 SLACs.

Typically, DyPs and LCMOs are only broadly classified by existing pHMMs, or in sequence databases. We therefore applied phylogenetic profiling with TreeSAPP [39] to classify these enzymes into discrete sub-families (Fig. 5A). Sequences from MAGs were placed only into A and B DyP types. Few DyPs were recovered (Fig. 4), none of which contained secretion signals. Nevertheless, DyP2, a C-type DyP from *Amycolatopsis* sp. 75iv2 involved in lignin depolymerization has no detectable signal sequence [36]. In contrast to DyPs, 82 LCMOs were detected in hot spring MAGs, and 100 were detected in hog fuel MAGs. Of these, only four were detected in LCCED sub-family 11, corresponding to SLAC or K-type laccases, all of which contained TAT signal peptides (Fig. 5B). Additionally, an O-type two-domain LCMO was detected in MB2.51 (*Steroidobacteraceae*), also containing a leading secretion signal peptide (Fig. 5C). We named this enzyme LacO_ST51_.

The classification of LCMOs into super-families, and comparison with enzymes of known function, may shed light on their functional roles. We applied structural alignment to assess the relationships between sequence, structure and function (Fig. 5D). Cu- and substrate-binding residues were highly conserved across K-type LMCOs including those recovered in this study. However, a 15-amino acid sequence hypothesized to act as a “flap” covering one of the channels leading to the trinuclear cluster [40], which was present in all known SLACs, was absent in the recovered thermophilic K-type LMCOs. Based on these sequence differences and the results of phylogenetic clustering, we categorize the LMCOs recovered from thermal systems as K2-type LCMOs, in contrast to the K1-type LCMOs found in mesophilic *Actinobacteria* such as *Streptomyces coelicolor* [40]. The lignin-degradation potential and thermotolerance of the K2 laccase clade remains uncharacterized.

### Putative tetrahydrofolate-dependent *O*-demethylases

**Fig. 6:**
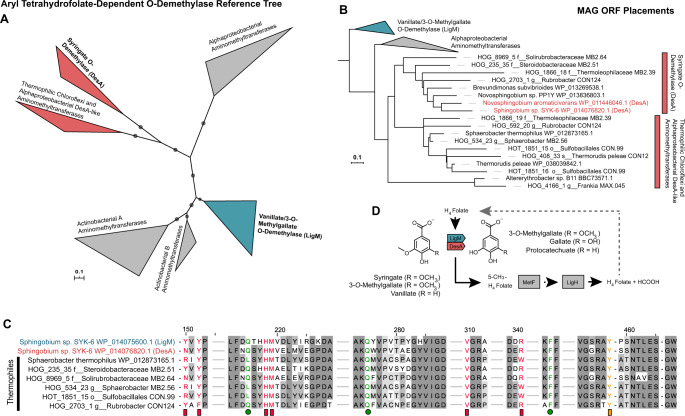
Aminomethyltransferase family protein phylogeny using TreeSAPP reference package. **A** Reference tree produced by TreeSAPP with 50 amino acid sequences using RAxML under the PROTGAMMALG model and 1000 iterations. Tree includes experimentally-validated vanillate/3-*O*-methylgalate *O*-demethylase (LigM) and syringate *O*-demethylase (DesA) proteins. **B** Placement of predicted MAG-encoded aminomethyltransferases into the reference tree. **C** Multiple sequence alignment with MAFFT using the *ginsi* setting under 1000 iterations for select sequences. Aromatic-binding residues derived from LigM structural model are shown in pink, folate-binding residues are shown in green, and the primary methyl-transferring catalytic tyrosine residue shown in orange. **D** Pathway diagram of tetahydrofolate-dependent O-demethylation of methoxylated aromatic compounds.

As our model lignin is comprised of 100% guaiacyl- subunits, we hypothesized that mineralization of DHP requires *O*-demethylation. While we identified a small number of vanillate *O*-demethylases (Fig. 4), which are Rieske-type oxygenases, we also investigated the potential for tetrahydrofolate-dependent *O*-demethylation of methoxylated aromatic compounds. To interrogate MAGs for tetrahydrofolate-dependent aryl *O*-demethylases, we once again used phylogenetic placement. We categorized aminomethyltransferases by putative function and taxonomic identity, with LigM and DesA sequences partitioning into distinct clusters (Fig. 6A). Assembled sequences placed into the tree formed a separate clade emerging from the DesA branch, which we have labeled “DesA-like aminomethyltransferases” (Fig. 6B). Specifically, MB2.64 (*Solirubrobacterales*), MB2.39 (*Thermoleophilaceae*), CON124 (*Rubrobacter* sp.), and MB2.51 (*Steroidobacteraceae*) all contained what appear to be DesA-like aminomethyltransferases, with conservation of a methyl-transferring tyrosine residue verified by structure-guided protein alignment (Fig. 6C). While the metabolic function of these enzymes requires validation, it is intriguing that they may facilitate the *O*-demethylation of LDACs in thermophilic bacteria.

### Characterization of LacO_ST51_

A key question that emerged from metabolic reconstruction of ^13^C-DHP-enriched MAGs following the microcosm study was the mechanism for the observed lignin depolymerization. In the above analysis we focused on the LCMOs as a possible answer. To test this hypothesis, and potentially identify novel biocatalysts, we selected four two-domain LCMOs (two K2-type, one O-type, one N-type) for heterologous expression, with the objective of evaluating their role in depolymerizing lignin (Table 1). Of these only “LacO_ST51_,” the O-type LCMO from MB2.51, proved soluble when expressed in *E. coli* (Table 1). Expression in *Rhodococcus jostii* RHA1 did not improve the solubility of the other proteins. SDS-PAGE indicated that the LacO_ST51_ protein was purified to >99% apparent homogeneity and had a molecular mass of ~36 kDa. Purified LacO_ST51_ exhibited the blue color typical of laccases, and had an absorption band at 620 nm, characteristic of a T1 blue copper site. The preparation had a molar copper content of 4.0 ± 0.2, indicating that the purified LacO_ST51_ was loaded with a full complement of copper.

**Table 1:** The specific activity of LacO_ST51_ and other bacterial laccases.

Name	Strain	Small laccase	ABTS (U/mg)	DMP (U/mg)
LacO_ST5_^a,b^	*Steroidobacteraceae* MB2.51	Y	1.46	0.03
LacN_TG59_^a^	*Thermogemmatispora* MB2.59	Y	–	–
LacK2_TH39_^a^	*Thermoleophilales* MB2.39	Y	–	–
LacK2_SR64_^a^	*Solirubrobacterales* MB2.64	Y	–	–
sLac^b^	*Amycolatopsis* sp. 75iv3	Y	1.19	0.21
SLAC^c^	*Streptomyces coelicolor*	Y	0.98	na^k^
Ssl1^d^	*Streptomyces sviceus*	Y	21.7	na
SLAC^e^	*Streptomyces coelicolor*	Y	8	na
GeoLacc^f^	*Geobacter metallireducens*	N	6.67	0.04
CotA^g^	*Bacillus licheniformis*	N	16	na
CotA^h^	*Bacillus* sp. HR03	N	0.15	na
LacM^i^	metagenome	N	2.4	2.1
ThioLacc^j^	*Thioalkalivibrio* sp. ALRh	N	0.65	na

^a^This study.

^b^Reactions at 25 °C. For ABTS: 20 mM sodium acetate (*I* = 0.1 M), pH 5.0. For DMP: 20 mM sodium phosphate (*I* = 0.1 M), pH 8.0.

^c^Sherif et al. [69]. Reactions at 60 °C. For ABTS: 50 mM sodium acetate, pH 4.0.

^d^Gunne and Urlacher [70]. Reactions at 25 °C. For ABTS: 50 mM McIlvaine’s buffer, pH 4.0.

^e^Dubé et al. [71]. Reactions at 25 °C. For ABTS: 2-(*N*-morpholino)ethanesulfonic acid (MES)–glycine buffer 0.1 M, pH 4.0.

^f^Berini et al. [41]. Reactions at 25 °C. For ABTS and DMP: 20 mM HEPES, pH 5.6.

^g^Koschorreck et al. [72]. Reactions at 25 °C. For ABTS: citrate/phosphate buffer pH 4.0.

^h^Mohammadian et al. [73]. Reactions at 25 °C. For ABTS: 100 mM phosphate buffer, pH 4.0.

^i^Ausec et al. [74]. Reactions at 25 °C. For ABTS: multi-component buffer (10 mM trizma base, 15 mM sodium carbonate, 15 mM phosphoric acid and 250 mM potassium chloride, pH 4.0. For DMP: same buffer, pH 5.0.

^j^Ausec et al. [75]. Reactions at 25 °C. For ABTS: 200 mM phosphate-citrate (McIlvaine), pH 5.0.

^k^Not available.

LacO_ST51_ utilized 2,2′-azino-di-(3-ethylbenzthiazoline sulfonate) (ABTS) and 2,6-dimethoxyphenol (DMP) as reducing substrates with specific activities of 1.46 and 0.03 U mg^−1^, respectively. The specific activity of LacO_ST51_ for ABTS was of the same order of magnitude as that reported for other bacterial laccases (Table 1). However, specific activity for DMP was lower than the value for most other reported laccases. The oxidation of DMP was optimal at pH 8, and the enzyme retained ~90% activity at pH 9. These values are higher than the average reported for other bacterial laccases [41], and match the optimal values described for alkaline laccases (e.g. [42, 43]). LacO_ST51_ retained >50% of its activity after 12 h when incubated at 55 °C. However, the half-life of LacO_ST51_ dropped significantly at 65 °C. Generally, the high pH preference and the thermal stability indicate that the enzyme is suitable for industrial applications.

**Fig. 7:**
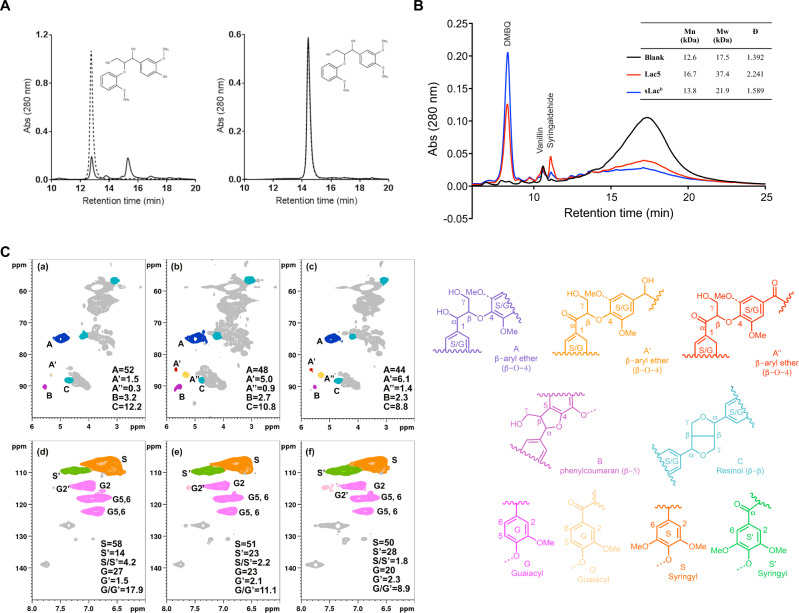
Transformation of lignin by LacO_ST51_. **A** Reactivity of LacO_ST51_with β-*O*-4 biaryl ethers. LacO_ST51_ (1 μM) was incubated for 6 h with 1 mM guaiacylglycerol-β-guaiacyl ether (left) or veratrylglycerol-β-guaiacyl ether (right) with 20 mM sodium phosphate and pH 8 at 55 °C. HPLC traces are of reactions with (solid line) and without (dotted) enzyme. **B** Treatment of EMAL with LacO_ST51_. LacO_ST51_ (6 μM) was incubated for 6 days with 0.5% (w/v) EMAL (12.5 mM sodium phosphate, pH 8, 10% DMSO, at 30 °C). HPLC traces are of reactions with (solid line) and without (dotted) enzyme. The identities of the indicated compounds were confirmed using authentic standards. DMBQ: 2,6-Dimethoxy benzoquinone. Inset: Effect of laccase treatment on molar mass distribution of Eucalyptus EMAL, where EMAL was treated with either LacO_ST51_, sLac or no enzyme (^b^sLac treatment generated insoluble material that was not analyzed using GPC). **C** HSQC NMR spectra of laccase-treated EMAL. EMAL was incubated with no enzyme (a) and (d), LacO_ST51_ (b) and (e), or sLac (c) and (f). The top and the bottom panels show the aliphatic and aromatic regions, respectively, of the 2D-NMR spectra. Linkages and units are expressed as per 100 aromatic units (100 Ar), which represented the integration of the G_2_ + 1/2S_2_. Structures of the regions are shown to the right.

To evaluate the ability of LacO_ST51_ to transform lignin, we initially tested the reactivity of LacO_ST51_ toward guaiacylglycerol-β-guaiacyl ether (GGE) and veratrylglycerol-β-guaiacyl ether (VGE), phenolic and non-phenolic compounds, respectively, that contain the β-*O*-4 linkage prevalent in lignin. GGE was depleted upon incubation with LacO_ST51_ for 6 h, and several products were detected by HPLC (Fig. 7A). The retention times of these compounds suggest that they are oligomerization products [44]. In contrast, the enzyme did not detectably transform VGE. These results indicate that LacO_ST51_ can react with phenolic substrates in the absence of mediators.

### Transformation of enzymatic mild acidolysis lignin by LacO_ST51_

We tested the ability of LacO_ST51_ to transform EMAL, a minimally altered form of lignin that contains little residual cellulose or hemicellulose from Eucalyptus wood [45]. A solution of EMAL incubated without enzyme for 6 days contained a significant quantity of vanillin (*t*_R_ = 10.6 min) and oligomeric material that eluted as a broad band (*t*_R_ = 13–22 min) (Fig. 7B). Incubation with 6 µM LacO_ST51_ additionally resulted in the production of syringaldehyde and 2,6-dimethoxy benzoquinone (DMBQ) as well as a reduction in the amount of oligomeric material. To provide insight into how LacO_ST51_ modified the lignin, the transformed lignin was isolated and characterized using NMR and GPC-MALS spectrometry. In these experiments, sLac was used as a positive control as it had previously been shown to transform lignin [7]. HSQC NMR spectra from laccase-treated lignin samples differed significantly from those of the no-enzyme controls (Fig. 7C). Specifically, in samples treated with either LacO_ST51_ or sLac, the secondary aliphatic hydroxyl groups of the lignin were oxidized to their benzylic ketone, as reflected by the increased in signals S’, G,’ A’, and A”. This oxidization process leads to the ability to cleave the propyl side chain with oxidative reagents resulting in rupture of native linkages (β-O-4, A) as illustrated with model compounds [46]. The slightly modified reaction on the C-C linkages including β-5 (B) and β-β (C) in our study and elsewhere [47] require more investigation. Based on the amount of linkages modified, sLac modified the lignin more efficiently than LacO_ST51_.

GPC analysis indicated that treatment of EMAL with either LacO_ST51_ or sLac yielded lignin with a higher apparent molar mass (M_w_ and M_n_) and increased the range of fragment size (Fig. 7B). However, sLac treatment resulted in a significant amount of insoluble material that was not included in this analysis. The higher molecular weight for both treated materials is presumably due to condensation reactions between the aromatic radicals [48]. The observed polymerization activity of LacO_ST51_ and sLac is consistent with studies of other laccases (e.g. [49]) and depends on the reaction conditions, particularly the relative concentrations of lignin species of different molecular weight. Indeed, sLac catalyzes the depolymerization of lignin in steam-pretreated poplar in the presence of natural mediators [7]. Overall, these data demonstrate that LacO_ST51_ oxidatively transforms lignin in the same manner as other laccases.

## Discussion

We hypothesized that lignin-degrading microorganisms occur in thermal environments that receive woody biomass inputs and would assimilate carbon from a synthetic lignin. The synthesis of ^13^C-DHP lignin in our laboratory was essential for accurate assessment of such assimilation, as ^13^C-labeled lignin of the purity used in this study is not commercially available. We verified lignin degradation in microcosms by monitoring ^13^C-CO_2_ production and ^13^C-incoropration into recovered DNA. The incorporation of ^13^C molecules from labeled, high-molecular-weight synthetic lignin into bacterial DNA provides direct evidence for bacterial catabolism of LDACs, and indirect support for bacterial lignin depolymerization. While it is evident that lignin was degraded, we cannot completely rule out other mechanisms of lignin depolymerization undetected by our methods. For example, a stable pool of extracellular fungal lignin peroxidases in our inoculum could have contributed to lignin depolymerization. However, genes putatively encoding lignin depolymerization were found in the MAGs of organisms that assimilated lignin in the microcosms, suggesting that thermophilic bacteria contributed to lignin depolymerization.

Several bacterial taxa were enriched with ^13^C from synthetic lignin. In hog fuel, these bacteria primarily belong to the proposed actinobacterial class *Thermoleophilia—*members of which are abundant in geothermal environments and soil but have poorly characterized metabolic potential [50]. Specifically, the genus *Rubrobacter* includes known thermophiles [51, 52], and *Rubrobacter* OTUs were strongly associated with lignocellulose degradation and tolerance of phenolic lignin metabolites at 55 °C [53]. Related actinobacterial MAGs, including from an *Actinomadura rubrobrunea* strain (CON22), were also ^13^C-enriched in hog fuel microcosms. CON22 contained one of few Rieske vanillate *O*-demethylases found in this study and encoded complete protocatechuate *ortho*-cleavage and partial *meta*-cleavage pathways (Fig. 4). While the lignin-degradation potential of *A. rubrobrunea* has yet to be characterized, other *Actinomadura* strains have been found to solubilize lignocellulose [54], and contribute to the degradation of the cellulose [55] or lignin [56]. The *Rubrobacter* and *A. rubrobrunea* strains identified in this study make compelling targets for further investigation. The MB2.64 MAG was placed in the actinobacterial family *Solirubrobacteraceae*. We previously identified putatively lignolytic *Solirubrobacterales* OTUs in forest soil through a similar stable isotope probing approach [14], but we were unable to resolve MAGs from lignin-assimilating *Actinobacteria* or identify enzymatic mechanisms that would explain their involvement in lignin degradation. We propose MB2.64 from hog fuel has robust lignin degradation potential. In contrast, in hot spring communities, ^13^C-enriched taxa were predominantly *Firmicutes* such as *Alicyclobacillus* ssp. Therefore, this study expands the taxonomic range of bacteria associated with lignin degradation to include other thermophilic *Actinobacteria* and *Firmicutes*.

In addition to incorporation of ^13^C from synthetic lignin and presence of putative ligninases, we used the presence of genes encoding catabolism of aromatic compounds to evaluate each MAG for its lignin degradation potential. A key difference between MAGs from hog fuel and hot springs was not only taxonomy, but also the capacity for aromatic catabolism. Hog fuel MAGs encoded vanillate *O*-demethylation and the protocatechuate *ortho*-cleavage pathway, suggesting that LDACs liberated from lignin were funneled into specific degradation pathways. Although several cultured representatives of *Solirubrobacteriales* encode a two-component Rieske vanillate *O*-demethylase (Fig. 4), none were found in the genome of MB2.64 from the hot spring. This was at odds with our hypothesis that *O*-demethylation is critical to catabolism of LDACs. However, a methyltransferase with full-length amino acid identity of 69.5% to syringate *O*-demethylase (DesA_SYK-6_), and with conserved substrate-binding residues, was identified through phylogenetic placement and sequence alignment. Accordingly, the 5,10-methylene-tetrahydrofolate reductase (*metF*) and formate-tetrahydrofolate ligase (*ligH*) genes, encoding the tetrahydrofolate-mediated C_1_ metabolic pathway [57, 58], were also found in the MB2.64 genome (Fig. 5D). Genomes of other Gram-positive bacteria such as *Rubrobacter xylanophilus* DSM9941 [57], *Acetobacterium dehalogenans* and *Desulfitobacterium hafniense* [59] (both *Firmicutes*) encode vanillate-demethylating methyltransferases. Thus, the MB2.64 genome provides strong evidence for lignin degradation mediated by bacterial thermophiles, facilitated by a novel one-component actinobacterial *O*-demethylase.

The ^13^C-enriched MAG, MB2.51, from hog fuel was placed in the gammaproteobacterial family, *Steroidobacteraceae*. As the name suggests, bacteria in this family can degrade steroidal hormones [60], but also polyvinyl alcohol [61] and rubber [62]. MB2.51 encoded full catechol and protocatechuate *meta*-cleavage pathways, as well as monooxygenases involved in 4-hydroxybenzoate and phenol hydroxylation. A methyltransferase from MB2.51 contained all conserved LigM_SYK-6_ residues involved in methyl-transfer, as well as aromatic substrate and folate binding, suggesting a role for this gene in degradation of methoxylated aromatic compounds. No thermophilic *Steroidobacteraceae* strains have been previously reported. Yet this MAG yielded the only soluble, thermotolerant laccase found in this study, discussed below.

Lignin degradation mechanisms in ^13^C-enriched hot spring MAGs are less clear than in their hog fuel counterparts. One possible explanation for this is cross-feeding, the catabolism by one organism of LDACs produced by a different lignin depolymerizing organism. However, the two ^13^C-enriched *Alicyclobacillus* sp. MAGs from hot spring sediment encoded a suite of L- and O-type LCMOs that bore high sequence identity to LCMOs found in *A. acidocaldarius* capable of non-specific cleavage of lignin-derived polyphenols [38]. The catechol *meta*-cleavage pathway genes in *Alicyclobacillus* sp. MAGs are incomplete, although the complete pathway is encoded in closely related *Alicyclobacillus* genomes (Fig. 4). We propose that, similar to *A. acidocaldarius*, the *Alicyclobacillus* sp. MAGs recovered herein encode non-specific oxidative degradation of lignin-derived polyphenols, which can serve as a source of carbon in carbon and nutrient limited oligotrophic ecosystems such as geothermal hot springs. This may also serve to as a detoxification mechanism.

Lignin-degrading organisms can serve as a source of novel ligninases, including laccases. Laccases are multi-copper oxidases that oxidize a broad range of compounds including substituted phenols, arylamines and aromatic thiols [63]. Bacterial laccases are appealing and versatile catalysts due to their thermal stability [64], use of molecular oxygen as the final electron acceptor and production of only water as a by‐product [65]. Here, we used newly published software, TreeSAPP [39], which places novel sequences into reference phylogenies. We designed a multi-copper oxidase reference phylogeny based on a model of 16 sub-families [66]. Specifically, we identified a possible thermophilic clade of the lignolytic two-domain K-type SLACs found in ^13^C-enriched *Solirubrobacteraceae* and *Thermoleophilales*, which we refer to as K2-type laccases. We also annotated a number of two- and three-domain LMCOs in ^13^C-enriched *Steroidobacteraceae* and *Alicyclobacillus* MAGs from sub-families O and L, which contain members capable of phenolic oxidation [67, 68]. Together, these results suggest that bacterial laccases are involved in lignin degradation in thermal environments.

To validate putatively lignin-degrading LCMOs identified with ^13^C-lignin SIP, we heterologously expressed a selection of these enzymes. Specifically, we attempted to express two K2-type, one O-type and one N-type laccase in *E. coli*. The two K2-type enzymes were expressed as insoluble forms, and expression of the N-type was not detected in either soluble or insoluble form. We also attempted to express the actinobacterial K2-type laccases in *Rhodococcus jostii* RHA1, but they were again insoluble—thus, the lignin degradation potential of the K2- and N-type laccases remains uncharacterized. The O-type laccase originating from the gammaproteobacterial MB2.51 MAG was expressed in soluble form in *E. coli* and further purified and characterized. This enzyme (LacO_ST51_) transformed a minimally transformed Eucalyptus lignin, liberating LDACs, including DMBQ and syringaldehyde. We previously demonstrated that syringaldehyde is a major degradation product of Eucalyptus lignin, and can be funnelled into the syringic acid *meta*-cleavage pathway in thermophilic *Alphaproteobacteria* [37]. Our results herein demonstrate that bacterial laccases, such as LacO_ST51_ or the previously-characterized sLac, can generate lignin-derived mono-aromatic compounds at elevated temperature. We propose that that these thermostable biocatalysts can be employed for bacterial bio-product production.

In this paper we characterized 14 genomes from bacteria in thermal hog fuel and hot spring sediment environments that incorporated synthetic ^13^C-labeled lignin. The results supported our hypothesis that these communities harbor thermophilic, lignin-degrading bacteria. These bacteria include members of the actinobacterial families *Solirubrobacterales* and *Thermoleophilaceae* that have a distinct clade of K2-type SLACs. These bacteria also included a *Gammaproteobacteria* (*Steroidobacteraceae*) from which we expressed a lignin-transforming O-type laccase. Overall, this study advanced our knowledge of how thermophilic bacteria can degrade lignin and LDACs and identifies enzymes potentially useful in biocatalysts for lignin valorization.

## Supplementary information

Supplemental Methods

Supplemental Data 1

Supplemental Data 2

## Data Availability

Sequence accessions are provided in Supplementary Data 1 and as part of NCBI BioProject PRJNA665309. Draft genome information and NCBI accessions are provided in Supplementary Data 2.
