# Supplementary material for: Discovery of lignin-transforming bacteria and enzymes in thermophilic environments using stable isotope probing
Source: ISME J. 2022 May 2;16(8):1944–56. doi: 10.1038/s41396-022-01241-8 (PMC9296663; doi:10.1038/s41396-022-01241-8)
Supplement: Supplementary file 1 — Supplemental Methods [file 41396_2022_1241_MOESM1_ESM.pdf]

Discovery of lignin-transforming bacteria and enzymes in thermophilic environments using stable isotope probing.

David J. Levy-Booth[1], Laura E. Navas[1], Morgan M. Fetherolf[1], Li-Yang Liu[2], Thomas Dalhuisen[1], Scott Renneckar[2], Lindsay D. Eltis[1], William W. Mohn[1*]

1. Department of Microbiology and Immunology, Life Sciences Institute, BioProducts Institute, The University of British Columbia, Vancouver, Canada
2. Advanced Renewable Materials Lab, Department of Wood Science, BioProducts Institute, The University of British Columbia, Vancouver, Canada

Running title: Probing for ligninases from thermophiles

[*]To whom correspondence should be addressed: William W. Mohn, Department of Microbiology and Immunology, Life Sciences Institute, The University of British Columbia, 2350 Health Sciences Mall, Vancouver, BC, V6T 1Z3, Canada. Tel.: +1- 604–822–4285; Fax: +1-604-822-6041; E-mail: wmohn@mail.ubc.ca

Supplemental Methods

Coniferyl alcohol synthesis

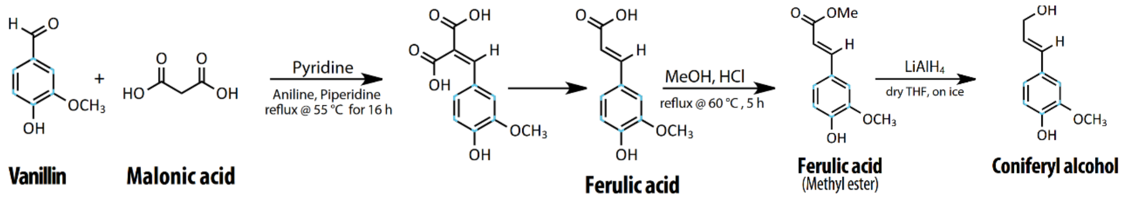

Figure SM1. Synthesis of coniferyl alcohol from vanillin and malonic acid. Blue atoms represent ^{13}C-labelled carbon. From [1].

Aldol condensation of vanillin and malonic acid to ferulic acid

Synthesis of ^{13}C-coniferyl alcohol used ring-labeled vanillin (75 atom % ^{13}C, Sigma Aldrich, St. Louis, U.S.A.). Synthesis of ^{12}C-coniferyl alcohol used unlabeled vanillin (99%, Sigma Aldrich).

Roughly 1:1 molar ratio of vanillin and malonic acid (1:1.2 if using ^{13}C-labeled vanillin), amounting to 2.5 g vanillin and 2.0 g malonic acid, were mixed in the presence of catalytic amounts of piperidine and analine (6 drops) in pyridine in a 2 neck round bottom flask (~25 mLs) and "refluxed" at 55°C for 16 h.

After 16 h, disappearance of vanillin was verified by thin-layer chromatography (TLC) in comparison to a standard to ensure reaction completion using 1:1 ethyl acetate:petroleum ether as the solvent. Upon completion, pyridine was removed using a rotary evaporator (RotoVap). Roughly 100 mLs of ethyl acetate was added to the mixture, which was then washed 3x with 1 M HCL. TLC was again used to ensure all product was located in the organic layer. Product was re-extracted with ethyl acetate if necessary. The solution was dried with $MgSO_4$ and the organic layer was recovered and the solvent was removed by RotoVap.

Esterification of reaction

Ferulic acid was dissolved in ~10-15 mL of MeOH. Roughly 50 ul of concentrated HCl was added, and the reaction was allowed to reflux for 4 hours to overnight. Progress was again monitored using TLC. Once the reaction reached completion, MeOH was removed using the RotoVap and the remaining product was dissolved in ethyl acetate, washed with H_2O, then wash again with

$NaHCO_3$. The remaining product was dried using $MgSO_4$ then the ethyl acetate was removed by RotoVap.

The compound was dry loaded onto a silica column (dissolve in MeOH and add silica to round bottom flask and rotovap out MeOH) for additional purification. The column was run using 1:1 ethyl acetate:petroleum ether as a solvent. The column was washed using a 3:2 ethyl acetate:petroleum ether, and pure ethyl acetate was used to elute the compound. The compound was verified using HSQC NMR and mass spectrometry.

Reduction of methyl ester

Reduction of the methyl ester to coniferyl alcohol is light sensitive and should be performed in the dark. Failure to do so results in the dimerization of coniferyl alcohol. A 1:1 molar ratio of $LiAlH_4$:compound was used for the reduction reaction. The reaction used a two neck round bottom flask with a large stir bar. The reaction was performed under positive N_2 pressure. Roughly 60 mL of freshly distilled tetrahydrofuran (THF) was slowly added to the $LiAlH_4$ while stirring in an ice bath. Next, the methyl ester was dissolved in ~14 mL of freshly distilled THF and added dropwise to the reaction. The reaction was kept on ice until all methyl ester was added. The add funnel was rinsed with ~10 mL of THF and added to the reaction. Following addition of all methyl ester, the flask was removed from the ice bath and stirring continued for ~2 hours. The reaction was monitored by TLC until the reaction was complete. Upon completion, the reaction was quenched by adding 12 mL ethyl acetate dropwise. Ethyl acetate must be added slowly at first, as the $LiAlH_4$ remains reactive. Next, 70 mL of 2 M HCl was added. Coniferyl alcohol was extracted 3x using 50 mL ethyl acetate, and washed once with 100 mL H_2O. TLC was used to confirm reaction completion and purity. Extraction with ethyl acetate was repeated if required. The ethyl acetate mixture was dried with $MgSO_4$, and solvent was removed by RotoVap.

A silica column was used to clean-up the coniferyl alcohol. It was first dissolved in dichloromethane (DCM) prior to loading, and eluted using 3:2 ethyl acetate:petroleum ether. Fractions were once again analyzed with TLC to ensure completeness and purity of the coniferyl alcohol product.

DHP synthesis

Coniferyl alcohol [1.7 g (9.45 mmol)] and 80 mg of peroxidase (HRP type II, Sigma-Aldrich, St. Louis, U.S.A.) were dissolved in 400 ml of degassed sodium phosphate buffer (100 mM) at pH 7.5. This solution and 9.45 mmol of H_2O_2 in 400 ml of degassed buffer were added

simultaneously and separately to 200 ml of well-stirred degassed buffer containing 20 mg of peroxidase and 30 mg (9.45 mmol) of vanillyl alcohol, over a 16 h period with a syringe pump (~ 0.4 ml/min). Coniferyl alcohol can be dissolved in a small volume (10-20 ml) of organic solvent (Acetone or DMSO) prior to addition to 400 ml buffer. All solutions were kept under N_2 and the reaction vessel was kept in dark (covered with the aluminum foil). After final addition, the mixture was stirred an additional 10 h. The insoluble polymer was separated by centrifugation at ~3000 x g for 30 minutes at 4°C and washed with deionized water (100 mLs). The washed polymer was freeze dried and stored at -20 °C.

DNA extractions and fractionation

DNA concentration was measured using the Qubit high-sensitivity DNA assay (Thermo Fisher Scientific, Waltham, U.S.A.). DNA was stored at -80 ^{o}C for up to five days prior to cesium chloride density gradient centrifugation and fractionation, according to published protocols [13,14]. Briefly, 1 ml gradient buffer (0.1 M Tris, 0.1 M KCl and 1 mM EDTA) containing 5μg DNA was mixed with 4.9 ml CsCl (1.878 g ml^{-1}) to a final density of 1.725 g ml^{-1} in 5.1 ml centrifuge tubes (Beckman Coulter, Brea, U.S.A). Density gradients were established over 42 hr at 44,100 rpm and 20 ^{o}C in an Optima L-90K ultracentrifuge (Beckman Coulter) with a Vti 65.2 vertical rotor (Beckman Coulter) in a vacuum, and without braking. Following centrifugation, a 23-gauge needle attached with tubing to a 60-ml syringe in an RE syringe pump (Razel Scientific, Saint Albans, U.S.A.) was used to pump water into the top of the tube at a rate of 0.5 ml min^{-1}. Twelve 0.42-ml fractions were collected dropwise in sterile 1.7-ml microtubes. The refractivity index (RI) of 20 μl of each fraction was immediately measured by an r^2 mini handheld refractometer (Reichert Technologies, Depew, U.S.A.), and density (D) in calculated using the equation:

(1) $D = (RI \times 10.927) - 13.593$

DNA in each fraction was immediately precipitated using 1 μl glycogen (Sigma-Aldrich) and 1.0 ml PEG6000 (Sigma-Aldrich), which was mixed, precipitated for 2 hr at room temperature, and centrifuged at 13,000 g at room temperature for 30 min. Supernatant was removed and the pellet was washed with 0.5 ml 70% ethanol. Following re-centrifugation as above, the ethanol was aspirated, the pellet dried for ~15 min and suspended in sterile 30 μl TE buffer (10 mM Tris-HCl, 1 mM EDTA, pH 8.0). The level of ^{13}C enrichment in each purified DNA fraction was quantified using ultrahigh-performance liquid chromatography-tandem mass spectrometry (UHPLC-MS/MS). Details are provided in [15] and in supplementary methods. This method allowed us to target sequencing of DNA fractions with higher confidence that originated from cells "enriched" by incorporation of ^{13}C-DHP.

Expression test of recombinant LMCOs

Genes encoding LCMOs were codon-optimized for *Rhodococcus jostii* RHA1 and synthesized by Twist Biosciences in the expression vector pET28a+, yielding pET_LacN$_{TG59}$, pET_LacK2$_{TH39}$, pET_LacK2$_{SR64}$ and pET_LacO$_{ST5}$. For expression in RHA1, genes were subcloned into pTipQC2. The nucleotide sequences of the constructs were verified.

Expression in *E. coli*: *E coli* BL-21 λ (DE3) transformants were grown overnight at 37 °C in LB supplemented with 50 mg l^{-1} of kanamycin. These cultures were used to inoculate 50 ml fresh LB medium with antibiotic to an optical density at 600 nm (OD_{600}) of 0.05. Cultures were grown to an OD_{600} ~0.6, amended with 0.5 mM IPTG, and incubated overnight at 30 °C. Control cultures with no IPTG were grown in parallel. Culture aliquots of 500 µl were collected and spun down. The cell pellet was resuspended in BugBuster Protein Extraction Reagent followed by an incubation of 60 min at RT. The supernatant of the lysate (soluble fraction) was removed and stored on ice. The remaining pellet was solubilized in buffer containing urea (50 mM Na-phosphate, pH 7.5, 300 mM NaCl, 8 M urea) and centrifuged. The solubilized supernatant ("insoluble" fraction) was removed and stored on ice.

Expression in RHA1: Freshly transformed RHA1 was grown at 30 °C in LB supplemented with 34 mg l^{-1} of chloramphenicol. These cultures were used to inoculate 50 ml fresh LB medium supplemented with antibiotic, to an optical density at 600 nm (OD_{600}) of 0.05. Cultures were grown to an OD_{600} ~0.8, induced with 5 µg ml^{-1} thiostrepton, and incubated for a further 24 h. Cells from 50 ml were suspended in 1 ml buffer (20 mM sodium phosphate, 300 mM NaCl, pH 8) containing protein inhibitor (cOmplete™, Mini, EDTA-free) and DNAse (2 µg ml^{-1}). Cells were subjected to five rounds of bead-beating at 5-6 m s^{-1} using a FastPrep®-24 (MP Biomedicals) with 5 min on ice between rounds. Cellular debris was removed by centrifugation. The supernatant of the lysate (soluble fraction) was removed and stored on ice. The remaining pellet was solubilized in buffer containing urea (50 mM Na-phosphate, pH 7.5, 300 mM NaCl, 8 M urea) and centrifuged. The solubilized supernatant ("insoluble" fraction) was removed and stored on ice. Presence of the recombinant LMCOs in fractions was assessed using SDS-PAGE.

References

1. Wilhelm RC, Singh R, Eltis LD, Mohn WW. Bacterial contributions to delignification and lignocellulose degradation in forest soils with metagenomic and quantitative stable isotope probing. ISME J. 2018; 1. doi:10.1038/s41396-018-0279-6
